# Intrinsic Fabry-Perot Interferometeric Sensor Based on Microfiber Created by Chemical Etching

**DOI:** 10.3390/s140916808

**Published:** 2014-09-10

**Authors:** Ruohui Wang, Xueguang Qiao

**Affiliations:** School of Physics, Northwest University, 229 Taibai Road, Xi'an 710069, China; E-Mail: xgqiao@nwu.edu.cn

**Keywords:** optical fiber devices, Fabry-Perot interferometers, temperature sensing

## Abstract

An intrinsic Fabry-Perot interferometeric sensor based on a microfiber has been demonstrated. The micro-size suspended core is created by chemical etching a photonics crystal fiber, of which the cladding has a micrometer-spaced, hexagonal array of air holes. The sensing head is fabricated by chemical etching a short section of photonics crystal fiber spliced with a single mode fiber. The temperature sensing characteristic of the interferometer has also been demonstrated and a sensitivity 14.3 pm/°C is obtained.

## 1. Introduction

Fiber Fabry-Perot interferometer (FPI), as a classic fiber optics component, has been widely used in various sensing applications for pressure, strain, temperature, and biomedical measurement [1]. Fiber Fabry-Perot interferometers are typically classified into two groups: extrinsic Fabry-Perot interferometer (EFPI) and intrinsic Fabry-Perot interferometer (IFPI). EFPI allows light to exit the fiber and propagate in an external cavity, while, in IFPI, light is restricted in the fiber. To form an FPI, it is essential to build two reflection mirrors, which could be interfaces between air and fiber, two fibers with refractive index mismatch or fiber and other waveguides with different refractive index. A typical EFPI, of which the cavity is an air gap, can be formed by inserting two cleaved optical fibers into a capillary tube [2]. Air gap based EFPI can also be fabricated by using Femtosecond laser micromachining techniques to create an in fiber notch [3]. Wafer [4] and thin film coating [5] have also been reported as cavities forming EFPIs. Typical EFPI cavities include graded index multimode fiber, capillary tube, and photonics crystal fiber [6–8] combined with single mode fiber. On the other hand, microfiber has attracted a great deal of attention in recent years due to its unique optical properties and applications in sensing fields. Microfiber is usually fabricated by heating and stretching or chemically etching a regular fiber to reduce its diameter to a few microns. The small size of the microfiber does not only bestow compactness, but also is largely evanescent filed and has a strong confinement, which make the microfiber a promising component in biochemical sensing and nonlinear effect generation. A large number of microfiber based sensing devices have been proposed, including microfiber Bragg grating, microcoil resonator, microfiber loop resonator, and Mach-Zehnder interferometer [9–12].

Recently, attempts to combine FPI and microfiber have also been reported. A challenge associated with fabricating FPI in microfiber is creating partial reflective mirrors in such small dimensions. It is difficult to transfer techniques of fabricating FPI to regular fibers to microfibers. Most of the reported configurations are IFPIs using FBGs as reflection mirrors. In 2005, Liang *et al.* [13] proposed the first microfiber FPI, based on an etch-eroded SMF between two FBGs as reflectors. Zhang *et al.* [14] later reported a similar FPI directly drawn from one normal FBG at its center by the flame-heated taper-drawing technique. Li *et al.* [15] directly inscribed a FPI in the uniform microfiber region by using 193-nm UV and exposed and analyzed its new characteristics, including the polarization dependence and large dispersion. In addition to grating-assisted structure, FP cavity in microfiber has also been formed by Sagnac mirrors, assembled with tellurite microfiber [16].

In this paper, we proposed a method to fabricate a microfiber-based IFPI by simply etching a short section of photonic crystal fiber (PCF) spliced to single mode fiber (SMF). The PCF has a pure silica cladding with hexagonal array of air hole structures. We use a hydrofluoric acid solution to erode the separations between the air holes and create a structure with a suspended core surrounded by the remained cladding. The micron-sized suspended core performs as the FP cavity and the two reflective mirrors are the interface between the SMF and suspended core and the fiber endface, respectively. The temperature-sensing characteristic of this microfiber FPI has been demonstrated. Such easy-fabrication and robust IPFI has the potential for many microfiber-based sensing applications.

## 2. Senor Fabrication and Principle

**Figure 1.**
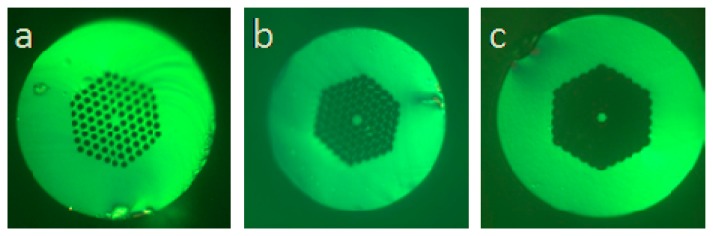
Evolution of the formation of the suspended core: Microscope photographs of the endless single-mode fiber (**a**) cross section view before etching; (**b**) cross section view after 40 s etching; (**c**) cross section view after 70 s etching. Figures 1a and 1c are reproduced with permission from [17]. Copyright 2014 Society of Photo-Optical Instrumentation Engineers.

Figure 1a shows the cross section view of the photonics crystal fiber (SM-7.0-PCF, Yangtze Optical Fiber and Cable Company) used in the experiment. The PCF is called endless single-mode fiber, which is originally designed for single-mode transmission over a wide wavelength range. It consists of a pure silica core surrounded by a photonics crystal cladding with a micrometer-spaced hexagonal array of air holes. The diameter of the fiber core and the air hole are 7 μm and 2.57 μm, respectively. The separation between two adjacent holes is 5.12 μm, which is smaller than the core size. Such dimensions enable us to create a suspended microfiber surrounded by the remained cladding. The fabrication process of the interferometer is simple and repeatable. A cleaved end of the PCF is spliced to a cleaved end of SMF using arc discharges. The splicing parameters, e.g., arc duration and arc power, need to be modified to repeat a splicing with smooth reflective interface and good mechanical strength. Fresnel reflection occurs at the splicing point due to refractive index difference between the doped SMF core and the pure silica PCF core. The spliced PCF is then cleaved to a short section with a desired length, L, with the assistance of a microscope. Afterwards, the fiber end was immersed in 49% hydrofluoric (HF) acid for about 120 s. When subjected to the chemical etching, the silica-based fiber core and the gaps between the holes are gradually eroded. The cross section view of the evolution of PCF during the etching process is presented in Figure 1. Since the separation between the adjacent holes is larger than the core size, the fiber core is no longer embedded within the periodic microstructure cladding and a suspended core structure is created after etching.

**Figure 2.**
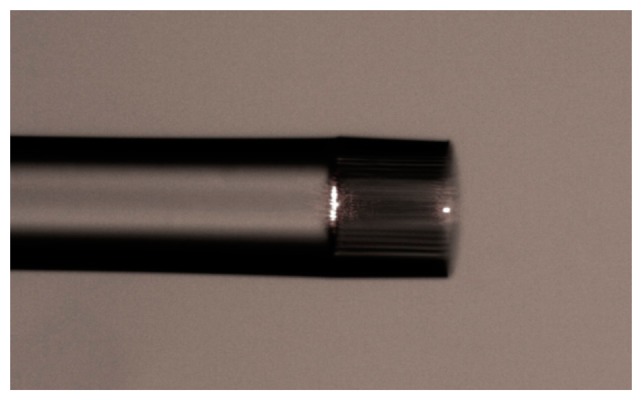
Microscope image of the sensor head illuminated with a He-Ne red laser.

**Figure 3.**
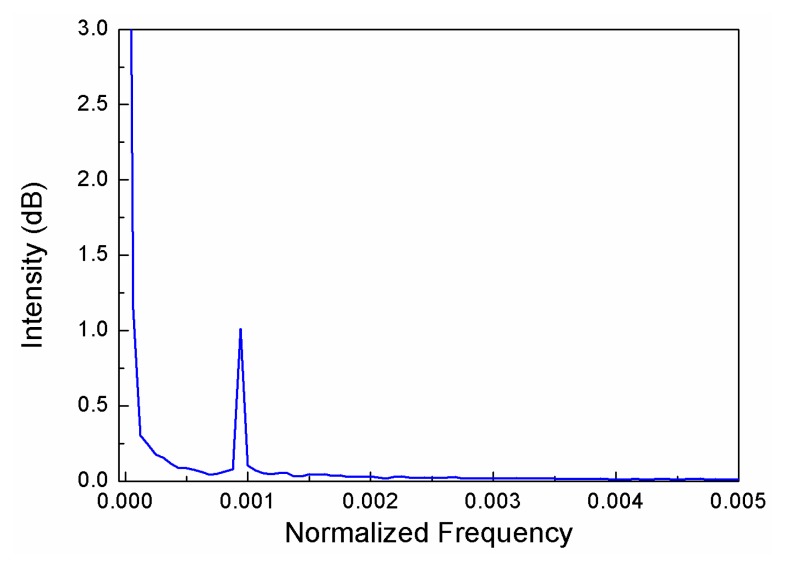
FFT results of the reflective spectrum of the IFPI.

The interference can be approximately explained as two-beam interference: light that propagates in the SMF is partially reflected at the interface between the SMF and suspended microfiber, but a fraction is coupled and travelling in the microfiber. This light is reflected when it reaches the end of the suspended core. Figure 2 shows the microscope image of the sensor head illuminated with a He-Ne red laser. Two bright spot indicating two reflections of the cavity, the splicing point (*R*_1_) and the end of the suspended core (*R*_2_), can be clearly observed. Figure 3 presents the Fast Fourier Transformation (FFT) of the interference pattern of a typical IFPI. The results indicated that only. The fundamental mode is excited and involved in the interference. The normalized reflective optical intensity *I* can be approximately expressed as:

(1) $$\begin{array}{ll} I & =R_{1}+{\left(1-A\right)}^{2}(1-R_{1})R_{2}+\\ & 2\sqrt{R_{1}R_{2}}(1-A)(1-R_{1})\cos(\frac{2\pi \text{OPD}}{\lambda }) \end{array}$$

where *R*_1_ and *R*_2_ are the reflection coefficients of SMF-microfiber and microfiber–air interface. *λ* is the wavelength in vacuum; *A* is the transmission loss factor at the SMF-microfiber interface. The transmission loss is attributed to the mode, mismatch between the lead-in SMF's fundamental mode field and the microfiber. OPD is the optical path difference and can be expressed as:

(2) $$\text{OPD}=2n_{\text{eff}}L$$

where *n_eff_* is the refractive index of the fundamental mode and the *L* is the cavity length.

When the interferometer is subjected to temperature measurement, the *OPD* change of fringe pattern can be given by:

(3) ΔOPD=OPD(α+ξ)ΔT

where α and ξ are the thermal expansion coefficient and thermo-optics coefficient, respectively. For the microfiber made of pure silica, α = 5.5 × 10^−7^ /°C and ξ = 8.3 × 10^−6^ /°C. The temperature change contributes refractive index and length variation of the cavity. The variations of the refractive index (RI) of the surrounding environment induce changes in the effective refractive index of the guided mode in the suspended fiber core. Therefore, the interferometer can be used for temperature and surrounding refractive index measurement.

## 3. Sensor Test Results

**Figure 4.**
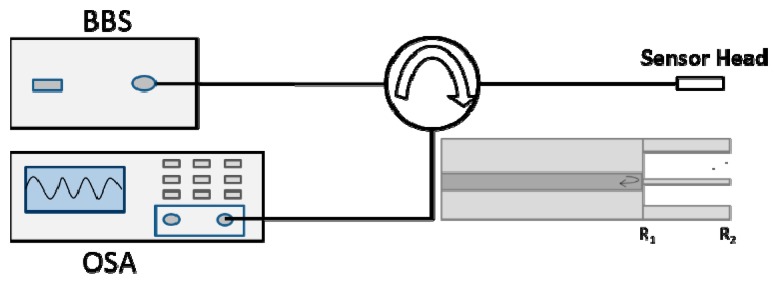
Experimental setup: inset is the schematic of the sensor head.

In the experimental setup, as shown in Figure 4, a C + L broadband light source (BBS) (Hoyatek, ASE-C + L module) is employed to illuminate the interferometer and the output spectrum is obtained by an optical spectrum analyzer (OSA) (YOKOGAWA AQ6370B). The sensor head was made by using a commercial fusion splicer (FSM-60S, Fujikura) in the manual mode. The micro FP cavity for performing the measurements had a length of ∼106 μm and diameter of the core is ∼2 μm, which was obtained after 120 s HF acid etching.

**Figure 5.**
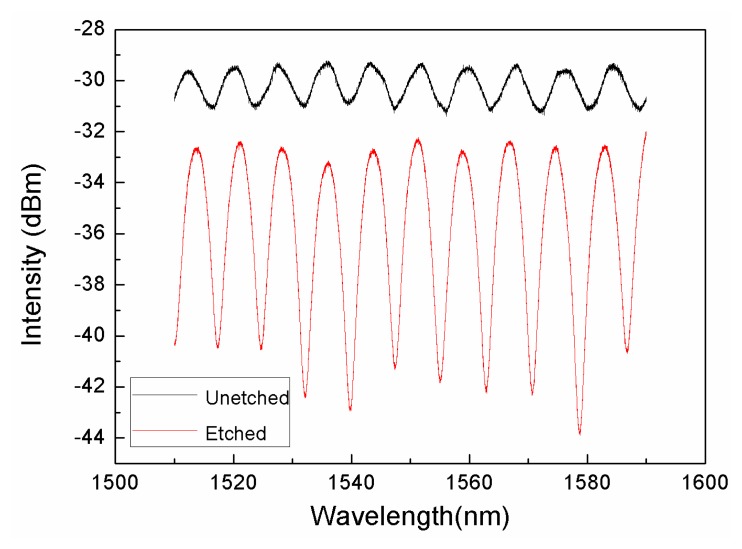
Interference patterns of IFPIs, based on unetched SMF-PCF (black line) and suspended microfiber (red line).

Figure 5 presents the typical interference pattern of the microfiber IFPI. The value of the IFPI's OPD can be roughly calculated as *OPD* = *λ*_1_*λ*_2_/2(*λ*_1_+*λ*_2_), in which *λ*_1_ and *λ*_2_ are wavelength of two adjacent peaks in the interference spectrum. The cavity length of this interferometer is calculated to be 106.2 μm, which is very close to the measured length using the microscope image. The fringe visibility is calculated to be 0.132, which is greatly improved compared with that of the SMF-PCF structure before etching (V = 0.029). The reduced fiber core size decreases the coupling efficiency at the interface and the reflection intensity at the fiber endface, which is reflected in the intensity drop in the spectrum. It is worth noting that no fluctuation of fringe pattern was observed during manipulation and transfer of the sensor within the laboratory. It is indicated that the microfiber is robust, even through its size is down to 2.2 μm with a length of 106.2 μm. To theoretically prove this, we treat the freestanding fiber as a cantilever and analyze its vibration behavior. The resonance frequency of a cantilever can be described as:

(4) $$f=\frac{1}{2\pi }\sqrt{\frac{8EI}{\rho Al^{4}}}$$

*E* is the Young's modulus of elasticity, *I* is the moment of inertia of the beam about the pivot, *ρ* is the mass density, *l* is the beam length. For the microfiber-based cylindrical beam, *A* = *πa*, where *a* is the radius of the fiber, and *I* = *πa^4^/4*. Typical values for the free-standing microfiber are *E* = 73 GPa, *ρ =* 2650 kg/m^3^, *a* = 1.1 μm and *l =* 106 μm. The calculated resonance frequency is up to 115.7 kHz. Therefore, the manipulation-induced vibration with a low frequency will not introduce bending in the microfiber. The interferometer is robust and portable compared to previously reported two FBGs-based FPIs [13], which require two holders to keep the stability the eroded fiber.

**Figure 6.**
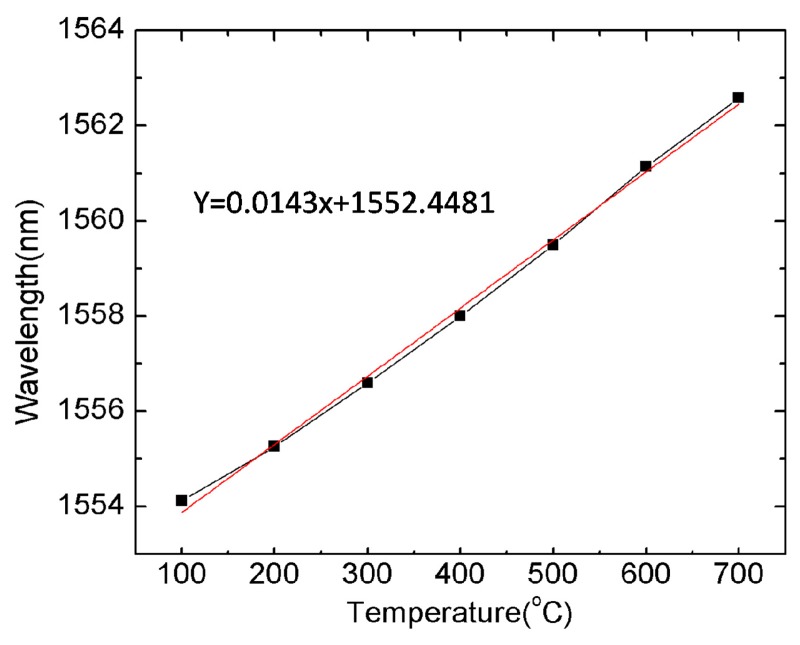
Wavelength shift of selected notch due to the temperature change. Reproduced with permission from [17]. Copyright 2014 Society of Photo-Optical Instrumentation Engineers.

We demonstrate the application of this IFPI as a temperature sensor. The sensor is positioned in a ceramic tube and then placed in a tube furnace for temperature measurement. The ceramic tube is employed to protect the sensor from contamination and to dampen the temperature fluctuation. The variation of the fringe pattern was recorded in the heating process from room temperature up to 700 °C with steps of 100 °C. The wavelength tracing method is used to interrogate the interferometer. Figure 6 presents the shift of a selected notch to longer wavelength as temperature increases. A temperature sensitivity of 14.3 pm/°C is achieved, which is higher than that of a typical fiber Bragg grating (FBG). Considering that the resolution of the OSA is 0.02 nm, the resolution of the IFPI is estimated to be 1.3 °C. The material of the PCF is pure silica, which has a melting point as high as 1670 °C. Therefore, our sensor has the capability of high-temperature measurement. While the standard FBG (type I) is not able to work in harsh environments (T > 450 °C) since the refractive index change is annealed out at these temperatures. In some applications, the FBG is too long to be conductive to small heating conductors. Our sensor has a compact sensing range of 106 μm in length, which is only two-hundredth of the FBG. Moreover, the fabrication of the IFPI-based sensor is simple and its cost is low, only involving fusion splicing and chemical etching.

**Figure 7.**
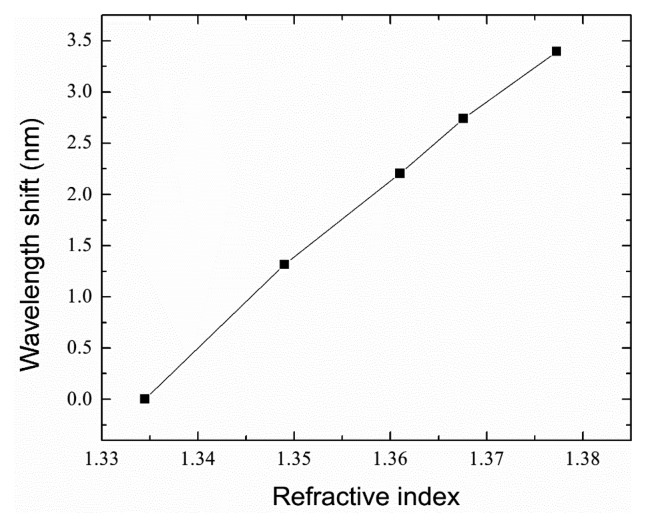
Wavelength shift as a function of refractive index changing.

To characterize its capability for refractive index sensing, the microfiber-based IFPI was subjected to different-concentration glycerol solutions, with RIs ranging from 1.334 to 1.337 at room temperature. The RIs of the different solutions are calibrated by an Abbe refractometer. The sensor head was immersed into each solution and the fringe pattern was recorded. Red shift of the reflective spectrum was observed and the wavelength of a selected resonance dip, as a function of RI, was shown in Figure 7. A sensitivity of 79.3 nm/RIU was achieved. This value is comparable with that of a microfiber-based FPI sensor formed by two FBGs [13], while our configuration shows a good robustness.

## 4. Conclusions

Summarizing, a microfiber-based Fabry-Perot interferometer was described. The FPI's cavity is a suspended core, obtained through a micromachining process, based on etching the endless single-mode fiber. The cladding with honeycomb air holes enable us to produce a unique structure consisting of a microfiber protected by the cladding. Since the fabrication process only involves fusion splicing, cleaving, and chemical etching, it presents a potentially cost-effective process and is able to be mass produced. The temperature response of the interferometer was investigated and a sensitivity of 14.3 pm/°C was achieved. The IFPI was also tested for liquid RI measurement and a sensitivity of 79.3 nm was obtained. It is foreseen that this IFPI could be applied as a temperature sensor and a microfiber based biochemical sensor.
